# Multilevel Regulation of Peptidoglycan Dynamics in Bacteria: From Molecular Mechanisms to Applied Perspectives

**DOI:** 10.3390/biom16050657

**Published:** 2026-04-28

**Authors:** Chang Dong, Juane Lu, Luyu Xie, Hao Wu, Jianjun Qiao

**Affiliations:** 1 State Key Laboratory of Synthetic Biology, School of Synthetic Biology and Biomanufacturing, Tianjin University, Tianjin 300354, China; 2 Zhejiang Research Institute of Tianjin University (Shaoxing), Shaoxing 312300, China; 3 Key Laboratory of Systems Bioengineering, Ministry of Education (Tianjin University), Tianjin 300072, China

**Keywords:** bacteria cell wall, peptidoglycan, regulatory mechanism, peptidoglycan synthase, peptidoglycan hydrolase

## Abstract

Peptidoglycan, a crucial constituent of the bacterial cell envelope, is essential for maintaining cellular integrity and morphology. Elucidating the regulatory processes that coordinate its biosynthesis and turnover not only addresses a fundamental question in microbiology but also reveals promising targets for antimicrobial drug development. This review summarizes recent advances in understanding the mechanisms governing peptidoglycan regulation, emphasizing the coordinated control of synthetic and hydrolytic pathways through multilayered networks that include transcriptional regulators, two-component systems, non-coding small RNAs, scaffold proteins, and protein–protein interactions. Building on these insights, we discuss the application of these regulatory principles in industrial biotechnology and the development of next-generation antimicrobial agents. Finally, we outline future research directions aimed at providing novel strategies to combat bacterial resistance and enhancing microbial platform engineering.

## 1. Introduction

The bacterial cell wall is a critical structural component required for maintaining cell shape and ensuring survival under environmental stress [1]. Its integrity is essential for withstanding osmotic pressure and preserving cellular stability [2]. The wall scaffold of both Gram-positive and Gram-negative bacteria consists primarily of the cross-linked polymer peptidoglycan [3]. This polymer is composed of long glycan chains of alternating β-1,4-linked N-acetylglucosamine (GlcNAc) and N-acetylmuramic acid (MurNAc) residues interconnected by short, flexible peptide bridges [4]. The chemical structure of these polysaccharide backbones is highly conserved across bacterial species. In contrast, the peptidoglycan of mycobacteria is additionally connected to an outer arabinogalactan-mycolic acid layer [5]. As the major component of the cell envelope, peptidoglycan undergoes continuous remodeling through synthesis and hydrolysis, processes that rely on a coordinated network of enzymes, scaffold proteins, and multi-enzyme complexes [6].

The biosynthesis of peptidoglycan is a highly complex and tightly regulated process [7,8,9,10] that involves the hydrolysis of peptidoglycan into fragments by hydrolases, followed by their transport into the cytoplasm and subsequent reuse in *de novo* synthesis [11]. Owing to its essential role in bacterial physiology, the mechanisms governing its synthesis and regulation remain a central focus of contemporary microbiological research. Recent progress has deepened our understanding of cell wall metabolism, including transcriptional, translational, and post-translational control mediated by transcription regulators, two-component systems, non-coding small RNAs, and scaffold proteins. Despite these advances, the precise mechanisms by which bacteria coordinate peptidoglycan synthesis with hydrolase activity remain insufficiently elucidated. To this end, the present study introduces the concept of “ multi-layered homeostatic regulatory architecture”. This term denotes the coordinated operation of at least three interdependent regulatory strata, namely the transcriptional, posttranscriptional, and posttranslational levels, which collectively maintain peptidoglycan synthesis within a physiological range while enabling the cell to respond rapidly to environmental or cell cycle cues.

Guided by this conceptual framework, this review focuses specifically on the regulatory mechanisms governing peptidoglycan metabolism. We begin by outlining the processes of peptidoglycan synthesis and hydrolysis, providing the necessary context for understanding their coordination. We then survey the diverse regulatory mechanisms, including transcriptional control, two-component systems, non-coding small RNAs, and scaffold proteins, that modulate these processes. Subsequently, we discuss how bacteria achieve homeostatic balance between synthesis and hydrolysis through integrated regulatory networks. We conclude by exploring the implications of these findings for improving the robustness of industrially relevant microorganisms and for informing the development of next-generation antibacterial agents.

## 2. Peptidoglycan Biosynthesis

### 2.1. Cell Wall Peptidoglycan Synthesis

**Figure 1:**
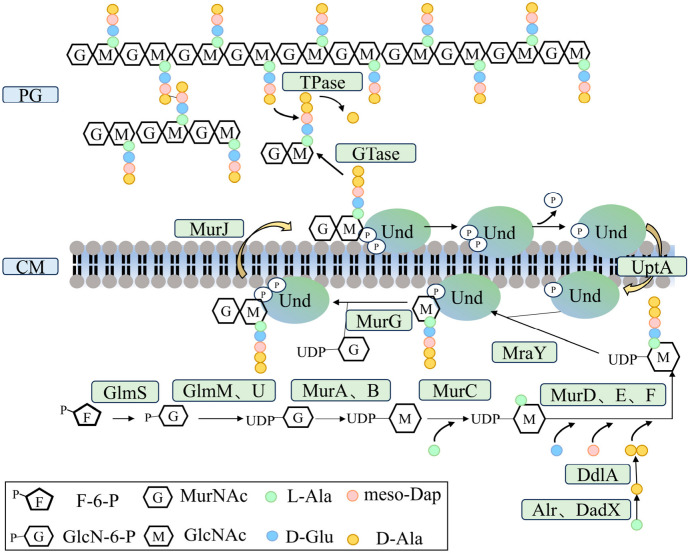
The formation of peptidoglycan precursors begins in the cytoplasm, where fructose-6-phosphate (F-6-P) is converted into UDP-GlcNAc through the Glm pathway. The enzymes MurA and MurB subsequently catalyze the transformation of UDP-GlcNAc into UDP-N-acetylmuramic acid (UDP-MurNAc). Subsequently, sequential addition of amino acids is catalyzed by the MurD-MurF enzymes, resulting in the formation of UDP-MurNAc-pentapeptide. The process then transitions to the cytoplasmic membrane, where MraY facilitates the formation of Lipid I by linking UDP-MurNAc-pentapeptide to undecaprenyl phosphate (UndP). The addition of UDP-GlcNAc generates Lipid II, which is translocated across the membrane by the flippase MurJ. On the extracellular surface, glycosyltransferases (GTases) and transpeptidases (TPases) catalyze the polymerization and cross-linking of glycan strands using Lipid II as a substrate, ultimately forming a new peptidoglycan layer within the cell wall matrix. Among these, l-Ala is converted into d-Ala–d-Ala (Alr and DadX) via the action of alanine racemase and d-alanyl–d-alanine ligase (DdlA). Extracytoplasmic UndP is transported back to the inner membrane by the undecaprenyl phosphate transporter UptA.

Peptidoglycan, the primary structural component of the bacterial cell wall, is composed of alternating N-acetylglucosamine and N-acetylmuramic acid residues cross-linked by short peptide chains, forming a mesh-like matrix. Its biosynthesis is a complex and highly regulated process that involves numerous enzymes and lipid carrier molecules [12]. Peptidoglycan assembly proceeds through three spatially and temporally coordinated stages (Figure 1).

(1) Cytoplasmic synthesis of precursors

The first stage occurs in the cytoplasm, where the enzymes UDP-N-Acetylglucosamine Enolpyruvyl Transferase (MurA), UDP-N-acetylglucosamine-enolpyruvate reductase (MurB), Mur ligases (MurC, MurD, and MurE) sequentially catalyze the formation of the UDP-activated precursor UDP-GlcNAc, leading to the production of UDP-MurNAc-pentapeptide, the complete peptidoglycan building block.

(2) Membrane-associated assembly and translocation

The second stage takes place at the cytoplasmic membrane and involves lipid carrier-mediated transport. The pentapeptide precursor forms a pyrophosphate linkage with undecaprenyl phosphate to generate Lipid I, which is subsequently converted into Lipid II through the addition of N-acetylglucosamine by the glycosyltransferase (MurG). The completed peptidoglycan subunit is then flipped from the inner to the outer leaflet of the membrane by translocases, such as MurJ or proteins of the FtsW family, preparing it for incorporation into the growing cell wall [12]. Recent studies have further revealed that the lipid II flippases MurJ and Amj are not essential in *Bacillus subtilis*; rather, the lethality associated with their mutation stems from a reduction in the availability of the lipid carrier undecaprenyl pyrophosphate (Und-PP). Overexpression of Und-PP synthase rescues this phenotype, suggesting the existence of an MOP-independent lipid II transport pathway and underscoring the critical importance of Und-PP homeostasis for peptidoglycan biosynthesis [13].

(3) Periplasmic polymerization and cross-linking

In the final stage, newly synthesized glycan strands are integrated into the existing cell wall matrix. Periplasmic penicillin-binding proteins (PBPs) catalyze the transglycosylation and transpeptidation reactions that cross-link the polymer chains, conferring mechanical strength and structural stability to the peptidoglycan network [14].

Although the basic structure of peptidoglycan (PG) is conserved, its chemical composition varies among different organisms. The composition of the short peptide differs slightly between Gram-negative bacteria (e.g., *Escherichia coli*) and Gram-positive bacteria (e.g., *Bacillus subtilis*); in the former, it is L-Ala–D-Glu–*meso*-diaminopimelic acid (*meso*-Dap)–D-Ala–D-Ala, whereas in the latter, *meso*-Dap is replaced by L-Lys. Notably, the peptidoglycan of *Mycobacterium* exhibits distinctive structural features. The peptidoglycan is covalently linked to arabinogalactan and mycolic acids, forming a complex outer cell wall layer. Uncommon LD-transpeptide bonds are present between peptidoglycan strands, and O-acetylation occurs on *N*-acetylmuramic acid residues. These modifications not only enhance cell wall stability but are also closely associated with resistance to β-lactam antibiotics, thereby establishing *Mycobacterium* as a key subject in studies on the regulation of drug-resistant pathogens [5]. These differences have been extensively documented in previous reviews [10].

### 2.2. Synthase of Peptidoglycan

Peptidoglycan cross-linking during cell wall biosynthesis is primarily catalyzed by two classes of synthases: (1) Class A penicillin-binding proteins (aPBPs), which possess both glycosyltransferase and transpeptidase activities, and (2) SEDS-bPBP complexes, consisting of SEDS family proteins with glycosyltransferase function paired with Class B penicillin-binding proteins (bPBPs) that act as transpeptidases [15].

PBPs are DD-peptidases that exhibit peptidyl transferase, carboxypeptidase, and endopeptidase activities [16]. Through transpeptidation (cross-link formation), endopeptidation (cleavage of existing bonds), and carboxypeptidation (removal of terminal D-alanine residues), PBPs modulate the architecture and mechanical integrity of the peptidoglycan network [14]. These enzymes are traditionally classified by molecular weight and function into high-molecular-weight PBPs (HMW-PBPs) and low-molecular-weight PBPs (LMW-PBPs) [16]. High-molecular-weight PBPs (HMW-PBPs) are further divided into two functional classes: class A PBPs possess both transglycosylase and transpeptidase activities and are primarily responsible for peptidoglycan chain elongation and initial crosslinking, whereas class B PBPs act exclusively as transpeptidases, participating in cell division through interactions with proteins such as FtsZ. In contrast, LMW-PBPs (Class C PBPs) function primarily as endopeptidases or carboxypeptidases, regulating cross-linking density, facilitating cell wall remodeling and repair, and contributing to cytokinesis and autolysis [17].

Meeske et al. demonstrated that *Bacillus subtilis* mutants lacking all known aPBPs remained viable, exhibiting normal morphology and peptidoglycan architecture despite delayed cell wall synthesis [18]. The SEDS family protein RodA, previously of unknown function, was identified as compensating for aPBP loss, as its overexpression restored normal growth [15]. Similarly, Taguchi et al. revealed that FtsW, a SEDS protein previously thought to function exclusively as a Lipid II flippase, also exhibits glycosyltransferase activity [19]. This enzymatic property is conserved across Gram-negative bacteria, including *Escherichia coli* [20]. Importantly, SEDS proteins collaborate with bPBPs that exhibit transpeptidase activity. For instance, FtsW forms a complex with PBP3 during septal peptidoglycan synthesis, while RodA associates with PBP2 to drive lateral wall elongation [21].

## 3. Hydrolysis and Recycling of Peptidoglycan

### 3.1. Hydrolases of Peptidoglycans

Peptidoglycan hydrolases are a diverse group of enzymes responsible for degrading the bacterial cell wall and play key roles in processes such as cell growth, division, and autolysis [22,23]. By cleaving glycosidic or peptide bonds within the peptidoglycan matrix, these enzymes maintain the dynamic equilibrium of cell wall remodeling. Peptidoglycan hydrolases are generally categorized as glycosidases or peptidases, depending on the specific bonds they cleave [23].

Glycosidases act on glycosidic linkages within the glycan backbone. For example, autolysin (N-acetylmuramidase) hydrolyzes the β-1,4 bond between GlcNAc and MurNAc, leading to cell lysis [24]. In contrast, lytic transglycosylases (LTs, e.g., Slt enzyme in *E. coli*) cleave the same β-1,4 linkage through a non-hydrolytic mechanism that converts MurNAc into 1,6-anhydro-MurNAc (anhMurNAc) via an intramolecular reaction [25].

Peptidases, by comparison, target amide linkages within the peptide stems of peptidoglycan. For instance, N-acetyl-MurNAc-L-alanine amidase releases peptide stems from the glycan backbone by hydrolyzing the amide bond between MurNAc and L-Ala [26]. The exopeptidase DD-carboxypeptidase removes the terminal D-Ala residue, converting pentapeptides into tetrapeptides [27]. Additionally, LD-carboxypeptidases cleave the bond between meso-diaminopimelic acid (*meso*-DAP) or L-lysine at position 3 and D-Ala at position 4, yielding tripeptides. Endopeptidases further process other amide bonds within peptide stems and interpeptide cross-links, contributing to peptidoglycan turnover and structural maintenance [28].

Acting as both “constructors” and “potential disruptors”, peptidoglycan hydrolases are essential for maintaining the structural balance of bacterial cell walls. By locally degrading the peptidoglycan matrix, these enzymes enable the incorporation of newly synthesized material and the formation of the division septum during cell proliferation [29]. However, their potent hydrolytic activity can become detrimental if not tightly regulated. Under environmental stress or antibiotic pressure, uncontrolled enzymatic action may lead to excessive wall degradation and trigger autolysis [30]. Because these enzymes embody both the regenerative force required for growth and the destructive potential that threatens survival, precise regulation is indispensable for preserving cell wall integrity.

### 3.2. The Cycle of Peptidoglycan Recycling

**Figure 2:**
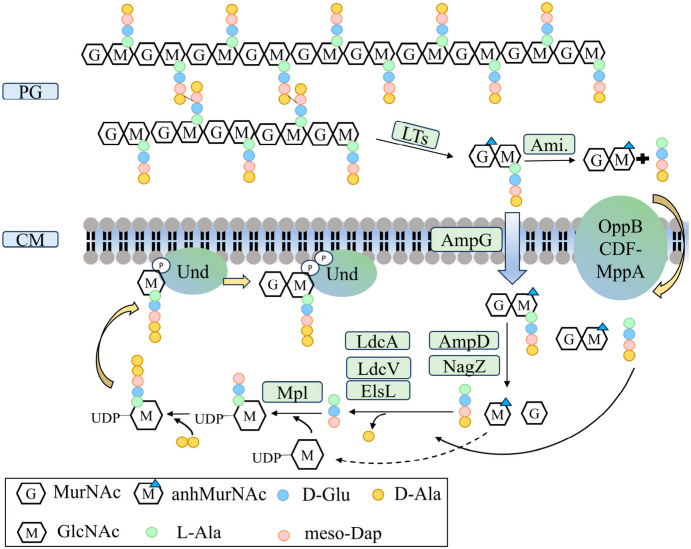
Schematic representation of peptidoglycan recycling in Gram-negative bacteria. In *Escherichia coli*, hydrolytic enzymes such as lytic transglycosylases (LTs) and amidases (Ami) degrade mature peptidoglycan, generating fragments composed mainly of anhydro-sugar derivatives and peptide chains. These degradation products are transported back into the cytoplasm via the AmpG permease or the OppBCDF-MppA ABC transporter system. Once internalized, AmpD (a cytoplasmic amidase) and NagZ (a β-N-acetylglucosaminidase) sequentially cleave peptide and glycosidic bonds to release free peptide fragments and sugar moieties. The peptides are then trimmed to tripeptides by LD-carboxypeptidases such as LdcA before being reattached to UDP-MurNAc by Mpl ligase, forming the UDP-MurNAc-tripeptide precursor required for peptidoglycan resynthesis.

Peptidoglycan hydrolases play a pivotal role in bacterial cell wall remodeling and division. During cytokinesis, they locally degrade the peptidoglycan matrix to enable the insertion of new wall material and the formation of the division septum. These enzymes are also implicated in autolytic processes; under antibiotic exposure or other adverse conditions, uncontrolled activity can cause excessive wall degradation leading to cell lysis. Moreover, the fragments generated by hydrolase-mediated cleavage can be transported into the cytoplasm and recycled into precursors for new cell wall synthesis, a process known as the peptidoglycan recycling cycle (Figure 2) [31].

In *Escherichia coli*, LTs primarily release peptidoglycan fragments containing anhMurNAc, whereas amidases (AmiA, AmiB, AmiC, and AmiD) cleave the amide bond between MurNAc and the attached peptide, liberating peptide chains. The major facilitator superfamily (MFS) transporter AmpG mediates the import of degradation products generated by endopeptidases and carboxypeptidases that sever peptide cross-links or shorten peptide stems [32]. Uptake of short peptide fragments is additionally facilitated by the OppBCDF-MppA ABC transporter system [33]. After anhMurNAc enters the cytoplasm, it is successively processed by AmpD (a cytoplasmic amidase), and NagZ (a β-N-acetylglucosaminidase). These enzymes cleave peptide and glycosidic linkages, respectively, releasing free amino acid fragments and monosaccharides [34,35].

To prevent cytotoxic accumulation and enable reuse via Mpl ligase, tetrapeptides are trimmed to tripeptides by LD-carboxypeptidases such as LdcA, LdcV, or ElsL during recycling [36]. If peptide fragments are not reincorporated into peptidoglycan precursors, they are further degraded by proteases such as MpaA or NlpC/P60 family enzymes (e.g., YkfC) into amino acids, which are subsequently metabolized by epimerase YcjG and deaminase DadA to serve as carbon and nitrogen sources [37]. In parallel, GlcNAc can be taken up and phosphorylated by the NagE phosphotransferase system, then funneled into central carbon metabolism following deacetylation by NagA and deamination by NagB [38]. MurNAc is imported via MurP or MurT and subsequently either phosphorylated and converted into UDP-MurNAc through the AmgK/MurU pathway or transformed into GlcNAc-6-phosphate by MurQ etherase, which removes the lactyl moiety [39,40].

Comparable recycling pathways are also present in Gram-positive bacteria such as *Bacillus subtilis* and *Staphylococcus aureus*, although their enzymatic components and regulatory mechanisms differ [41]. Its NagZ may be localized either extracellularly or in the cytoplasm, and during internalization, owing to the absence of an AmpG homolog, it primarily relies on ABC transporters (e.g., DppBCDF-YkfD) [42]. Hydrolysis of peptidoglycan-derived sugars by extracellular NagZ and NamZ releases monosaccharides that are subsequently recovered through analogous enzymatic reactions. The resulting fragments may also modulate the expression of PBP2a or β-lactamase via the BlaR1/BlaI signaling pathway [43,44]. Peptidoglycan recycling thus plays a crucial role in the long-term viability and adaptability of Gram-positive species [45].

## 4. Regulatory Mechanisms of Peptidoglycan Biosynthesis and Hydrolysis

The coordination between peptidoglycan synthesis and hydrolysis forms the foundation of bacterial cell wall homeostasis. As discussed above, these two processes involve a series of enzymatic reactions, and the spatiotemporal activities of these functionally opposing enzyme families must be maintained in exquisite balance. Any disruption to this balance may lead to aberrant cell wall thickening or, more critically, catastrophic cell lysis. Achieving such balance requires the integration of multiple regulatory signals that modulate the expression and activity of these enzymes.

In the following sections, we explore the specific mechanisms that finely regulate peptidoglycan metabolism. We first focus on specific transcription factors that directly bind to the promoter regions of target genes. We then discuss how two-component systems, as canonical signal transduction modules, convert extracellular signals into transcriptional outputs. In addition to these classical regulators, emerging studies have highlighted the critical roles of non-coding small RNAs (sRNAs), scaffold proteins, and protein–protein interactions in maintaining cell wall peptidoglycan homeostasis, primarily acting at the post-transcriptional level and adding further layers of refinement to the regulatory network. Importantly, these regulatory layers do not function in isolation; rather, they act in concert through coordinated and feedback mechanisms at the genetic, transcriptional, and translational levels to collectively maintain peptidoglycan homeostasis. Together, these multilayered regulatory mechanisms ensure the precise coordination of peptidoglycan synthesis and hydrolysis, allowing cells to preserve cell wall integrity under fluctuating environmental conditions.

### 4.1. Transcription Regulator

Transcriptional regulators bind selectively to specific promoter sequences, thereby modulating gene expression in a spatially and temporally controlled manner [46]. Their distinct structural and functional properties allow them to modulate transcriptional efficiency by specifically activating or repressing target downstream genes [47]. Within bacterial cell wall regulatory networks, these factors precisely coordinate the expression of genes involved in peptidoglycan metabolism. This transcriptional control is closely associated with the activity of RNA polymerase [48], their precise coordination ensures that expression of peptidoglycan associated genes remains within the basal physiological range, representing the most classical and most extensively investigated layer within the multi-layered homeostatic regulatory architecture.

**Figure 3:**
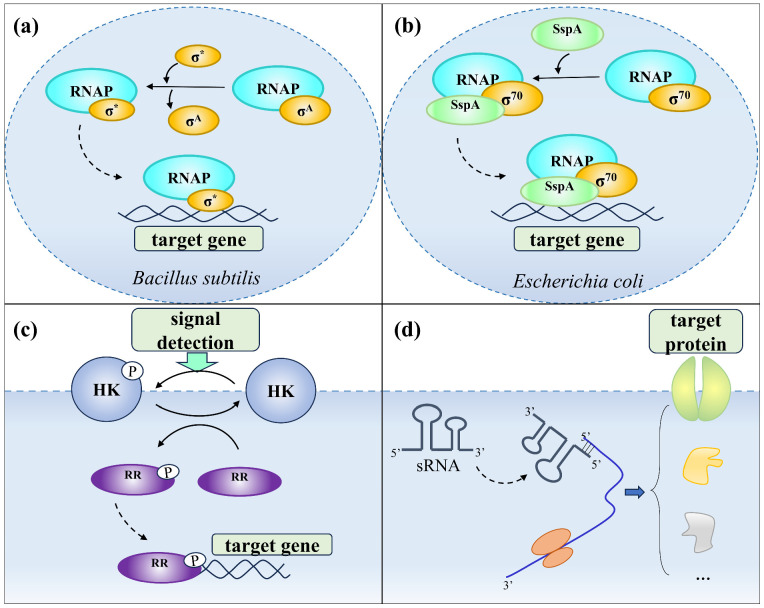
Regulatory systems governing bacterial cell wall peptidoglycan synthesis. (**a**) Gram-positive regulatory network: Alternative σ factors (σ*) replace the σ^A^ subunit within the RNAP holoenzyme complex, altering transcriptional activity and directing the expression of specific gene sets. (**b**) Gram-negative regulatory network: The stringent starvation protein SspA interacts with the σ^70^-RNAP complex to modulate transcriptional responses. (**c**) Two-component signaling pathway: The system comprises a histidine kinase (HK) and a response regulator (RR) that function through a “signal detection-phosphoryl transfer-gene regulation” cascade. (**d**) sRNA-mediated regulation: Non-coding small RNAs (sRNAs) influence cellular processes by modulating target gene expression at the post-transcriptional level, thereby contributing to the control of peptidoglycan metabolism.

In Gram-positive bacteria, alternative sigma (σ) factors serve as key transcriptional regulators. For instance, *Bacillus subtilis* encodes 17 distinct σ factors that substitute for the housekeeping σ^A^ subunit within the RNAP holoenzyme, thereby redirecting transcription toward specific gene sets (Figure 3a; Table 1). Among them, σ^M^ plays a central role in maintaining cell wall integrity and regulating multiple stages of peptidoglycan synthesis. It activates genes encoding structural proteins such as PBP1 and PBPX involved in cell wall assembly, as well as enzymes including *ddl*, *murB*, and *murF*, which participate in precursor formation [49]. Additionally, σ^M^ controls genes associated with morphogenetic complex formation (*mreBCD*, *rodA*, *divIB*, *divIC*, and *minCD*) and the lipid II translocase AmJ, ensuring proper substrate transport during peptidoglycan biosynthesis [50].

Under stress conditions such as heat shock, σ^I^ assumes regulatory control. Deletion of this factor leads to abnormal, deformed cell morphology due to the marked downregulation of LytE, a peptidoglycan hydrolase, and MreBH, a cytoskeletal protein [51,52]. Moreover, σ^I^ can enhance morphogenetic complex activity to partially compensate for σ^M^ deficiency [52]. Other σ factors, including σ^D^, σ^W^, σ^X^, and σ^V^, also modulate the transcription of specific synthase and hydrolase genes involved in cell wall metabolism [53,54].

Beyond σ factor-mediated regulation, additional transcription regulators contribute to peptidoglycan homeostasis. Bouillaut L et al. demonstrated that DdlR, a GntR family regulator, directly binds the promoter of the *ddl* gene to ensure proper expression of D-alanine-D-alanine ligase, a key enzyme in the peptide cross-linking reaction [55]. Furthermore, Wu et al. report that the global regulator CodY positively influences the transcription of *murA*, *murC*, and *murD*, with deletion of *codY* resulting in pronounced alterations in cell shape, wall thickness, and rigidity [56].

In Gram-negative bacteria, the stringent starvation protein SspA functions as a transcriptional regulator by binding to RNA polymerase and interacting with the σ^70^-RNAP complex [57]. This highly conserved factor is induced during the stationary phase or under nutrient-limiting conditions (Figure 3b; Table 1). Deletion of the *sspA* gene leads to elevated expression of PBP1B and SEDS-bPBP family genes, which compensates for peptidoglycan degradation caused by PBP1A deficiency. According to Lou et al., SspA in *Escherichia coli* coordinately regulates both classes of peptidoglycan synthase genes, suggesting that its repressive control of cell wall biosynthetic gene expression is a conserved mechanism among Gram-negative bacteria [58].

Additional transcriptional regulators also influence peptidoglycan homeostasis. Freire et al. demonstrated that overexpression of BolA, a stress response protein in *Escherichia coli*, induces morphological conversion from rod-shaped to spherical cells [59]. This phenotype arises from BolA-mediated repression of the *mreBCD* operon and concurrent activation of peptidoglycan hydrolase genes such as *dacA*, *dacC*, and *ampC*, thereby reshaping the cell wall through bidirectional regulation of gene expression.

A comparison of the above findings reveals both commonalities and notable differences in the peptidoglycan regulatory strategies employed by Gram-positive and Gram-negative bacteria. Gram-positive bacteria utilize alternative sigma factors as core regulators, establishing a regulatory network that spans the entire process of peptidoglycan metabolism. In contrast, Gram-negative bacteria tend to rely on global or stress-responsive transcription factors (e.g., SspA and BolA) to mediate transcriptional repression of peptidoglycan synthase genes and to modulate cell morphology. Although the regulatory architecture in Gram-negative bacteria appears relatively streamlined, it still retains cross-compensation capacity. Despite the divergence in the types and modes of action of regulatory factors between Gram-positive and Gram-negative bacteria, the core principle fine-tuning the balance between peptidoglycan synthesis and hydrolysis through transcriptional regulation is highly conserved across bacterial species.

**Table 1:** Upstream Transcriptional Regulation of Peptidoglycan Homeostasis.

Name	Category	Role	Model Organisms	References
Transcriptional regulators				
σ^M^	Alternative sigma factor	Maintaining cell wall integrity and regulating multiple stages of peptidoglycan synthesis	*B. subtilis*	[49]
σ^I^	Alternative sigma factor	Upregulating the expression of the MreBH complex protein and the peptidoglycan hydrolase LytE	*B. subtilis*	[52]
DdlR	GntR-family transcription regulator	Directly combined with the promoter of the D-alanine-D-alanine ligase gene *ddl*	*C. difficile*	[55]
CodY	Transcription regulatory factor	Regulates the PG synthesis genes *murA*, *murC*, and *murD*	*L. lactis*	[56]
SspA	RNAP-associated regulatory protein	Binding to RNA polymerase and interacting with the σ^70^-RNAP complex	*E. coli*	[57]
BolA	Transcriptional regulator	Repress mreBCD and activate hydrolase genes to remodel the cell wall	*E. coli*	[59]
Two-component System				
WalKR (YycFG)	Two-component system	Regulate peptidoglycan metabolism to ensure proper cell division and homeostasis	*B. subtilis*	[60]
AirSR	Two-component system	Activate cell wall biosynthesis genes	*S. aureus*	[61]
VxrAB (WigKR)	Two-component system	Upregulate the expression of genes related to the peptidoglycan synthesis pathway	*V. cholerae*	[62]
Cpx	Two-component system	upregulating the LD-transpeptidase gene *ldtA*	*E. coli*	[63]

### 4.2. Two-Component System

The two-component system represents the primary signaling mechanism through which bacteria sense and respond to environmental stimuli [64]. It comprises a histidine kinase (HK) and a cognate response regulator (RR) that function through a canonical cascade of signal detection, phosphotransfer, and transcriptional regulation. Upon perceiving external cues such as antibiotic stress or peptidoglycan damage, the HK undergoes autophosphorylation and subsequently transfers the phosphate group to its cognate response regulator [65]. The phosphorylated regulator then modulates the transcription of downstream genes, enabling adaptive responses to environmental fluctuations. This regulatory pathway plays a critical role in maintaining peptidoglycan homeostasis and coordinating cell wall remodeling (Figure 3c; Table 1). In the multi-layered homeostatic regulatory architecture, the two component system functions as a pivotal entry point that couples external perturbations to internal transcriptional adjustments: it converts physicochemical signals into phosphorylation signals, thereby mobilizing the network of transcriptional regulatory factors and enabling adaptive reprogramming of gene expression for the dynamic maintenance of peptidoglycan homeostasis.

The WalKR (YycFG) two-component system in *Bacillus subtilis* serves as the principal regulator of peptidoglycan metabolism in Gram-positive bacteria. Its regulatory network encompasses genes involved in cell division (such as the *ftsAZ* operon) and cell wall hydrolases (*yocH*, *cwlO*, and *lytE*). By modulating the transcription of these targets, WalKR ensures proper cytokinetic progression and maintains peptidoglycan homeostasis [60,66].

Similarly, in *Staphylococcus aureus NCTC8325*, the AirSR system positively controls the expression of more than 20 genes associated with cell wall biosynthesis [61]. Sun et al. demonstrated that AirSR directly binds to the promoter regions of key genes, including *cap*, *pbp1*, and *ddl*, which are integral to peptidoglycan synthesis [67]. Deletion of *airSR* results in pronounced autolysis, confirming its essential role in preserving cell wall integrity.

In Gram-negative bacteria, the VxrAB (also known as WigKR) two-component system of *Vibrio cholerae* plays a pivotal role in regulating peptidoglycan biosynthesis. Overexpression of the response regulator mutant VxrB^D78E^, which mimics the phosphorylated form, induces upregulation of multiple genes involved in cell wall assembly. These include those related to Lipid II translocation (*murJ*), polymerization and cross-linking (*ftsW*, *pbp1a*, *pbp1b*), and precursor formation (*murA-E*, *ddlA*, *mraY*, *murG*) [62]. Interestingly, VxrB directly associates with the promoter regions of *pbp1a* and *murJ*, although these binding motifs are not conserved, suggesting the involvement of additional cofactors in promoter recognition [68]. Notably, the VxrAB system appears to be restricted to the *Vibrionaceae* family. While a homologous pair, VbrKR, in *Vibrio parahaemolyticus* has not yet been confirmed to regulate cell wall synthesis, it has been shown to enhance β-lactamase gene expression, contributing to β-lactam antibiotic resistance [69].

Because the peptidoglycan layer of Gram-negative bacteria is enclosed by the cell wall, its synthesis is closely regulated by several two-component systems that monitor and respond to membrane stress. For example, in *Escherichia coli*, damage to the cell wall activates the Cpx signaling pathway, which alters peptidoglycan composition by upregulating the LD-transpeptidase gene *ldtA*, thereby preventing cell lysis [63]. Although the downstream regulatory mechanisms of these systems have been characterized, the specific signals detected by their HKs remain unclear. Current evidence is largely speculative, suggesting that WalK and VxrA may sense stimuli derived from yet unidentified peptidoglycan components.

### 4.3. Non-Coding Small RNA (sRNA)

Non-coding small RNAs (sRNAs) are short RNA molecules, typically 50–500 nucleotides in length, that lack protein-coding capacity [70]. They enable bacteria to fine-tune gene expression and adapt to environmental fluctuations by modulating various regulatory processes, including target protein activity, mRNA stability, and translational efficiency [71]. These molecules enable more rapid and energetically economical fine tuning of gene expression at the post transcriptional level. Within the multi-layered homeostatic regulatory architecture, the post transcriptional layer fills the temporal gap caused by the delayed response of the transcriptional layer and plays a critical role in maintaining cell wall homeostasis and coordinating the dynamic balance of peptidoglycan metabolism (Figure 3d; Table 2).

In *Escherichia coli*, the sRNA *glmZ* directly regulates the synthesis of UDP-N-acetylglucosamine by base-pairing with glmS mRNA, thereby activating *glmS* expression and ensuring a stable supply of precursors for cell wall biogenesis [72]. Cayrol et al. demonstrated that sRNA *dsrA* binds to the 5′ untranslated region (UTR) of *mreB* mRNA, reducing intracellular MreB protein levels by interfering with transcription and destabilizing the mRNA’s secondary structure, ultimately altering cell morphology [73]. Similarly, the Hfq-dependent sRNA *dicF* inhibits *ftsZ* translation by pairing with its ribosome-binding site, thereby affecting Z-ring formation during cytokinesis [74].

In *Listeria monocytogenes*, the Lmo0514 protein is essential for cell wall assembly, recognizing the LPXTG motif of a sorting protease and covalently anchoring to the cell wall through its activity [75]. The sRNA *rli27* stabilizes the 5′-UTR of *lmo0514* mRNA, thereby promoting Lmo0514 synthesis and fortifying cell wall resilience under stress conditions [76]. Despite these findings, the broader mechanisms by which sRNAs regulate peptidoglycan biosynthesis remain poorly understood. Further studies are required to elucidate the complexity and specificity of this regulatory network.

### 4.4. Scaffold Protein

Scaffold proteins play crucial structural and functional roles in bacterial cell wall regulation. Their main function is to maintain the precise spatial localization and functional stability of associated enzymes and protein complexes by providing defined binding sites through their stable architectures [86,87]. By coordinating the spatial distribution of peptidoglycan synthases and hydrolases, these organizers preserve the integrity and dynamic organization of the peptidoglycan network during cell growth and division. In the multi-layered homeostatic regulatory architecture, the posttranslational layer constitutes the structural foundation that ensures transcriptional and posttranscriptional instructions are executed at the correct time and in the correct location. Five major scaffold protein families, including MreB, FtsZ, GpsB, DivIVA, and EzrA, are recognized for their distinct contributions to cell wall regulation. Each family operates via specialized structural attributes and functional mechanisms, collectively supporting cellular morphogenesis (Table 2).

(1) MreB

MreB is a key scaffold protein responsible for sensing cell shape, promoting morphological changes, and maintaining structural stability [77]. It plays a central role in determining bacterial cell dimensions and morphology through a self-organizing feedback mechanism that ensures uniform cell architecture [88]. The number of MreB homologs varies among species: *Escherichia coli* possesses a single MreB protein, whereas *Bacillus subtilis* expresses multiple homologs that act redundantly to regulate cell shape. Additionally, loss of MreB function results in the loss of rod-shaped morphology [89].

Because the *Escherichia coli* cell wall resides in the periplasmic space, MreB coordinates peptidoglycan synthesis through transmembrane linker proteins that connect cytoplasmic scaffolds to periplasmic enzymes. The key linkers, MreC, MreD, and RodZ, bridge the MreB cytoskeleton to peptidoglycan synthases, with their cytoplasmic domains binding MreB and their periplasmic regions interacting with the synthetic machinery. This connectivity regulates cell shape by influencing MreB’s circumferential movement. Moreover, MreB localization is sensitive to surface geometry; it preferentially associates with regions of specific curvature, directing peptidoglycan insertion to maintain alignment between cell wall synthesis and overall morphology [90].

(2) FtsZ

The tubulin homolog FtsZ is a key cytoskeletal protein that orchestrates bacterial cell division. In the metaphase of cell division, FtsZ polymerizes into a ring-like scaffold known as the Z-ring, which recruits over ten division-related proteins to form the contractile machinery required for cytokinesis [79]. During the early stage of *Escherichia coli* division, FtsZ interacts with FtsA and ZipA at the future septation site, establishing a dynamic Z-ring at the cell midpoint [91]. The interaction between FtsA and ZipA further stabilizes this structure, maintaining its integrity throughout the division process [92]. The mature Z-ring also serves as a platform for the recruitment of MurG and other peptidoglycan synthases, thereby coupling septal wall synthesis with constriction and enhancing the efficiency of peptidoglycan assembly [93].

(3) EzrA and GpsB

In Gram-positive bacteria, the scaffold proteins GpsB and EzrA are highly conserved and act cooperatively to coordinate cell wall biogenesis [94]. GpsB is a hexamer, while the isolated N- and C-terminal domains are dimeric and trimeric, resulting in a tripod-like conformation [95]. This structure enables simultaneous interaction with multiple membrane-associated PBP4 molecules, thereby influencing their spatial organization during cytokinesis and regulating peptidoglycan assembly [96]. The latest research by Bhattacharya et al. confirms that the GpsB interacts with FtsZ in multiple species and may serve as an accessory Z-ring anchor [97]. EzrA, a multifunctional scaffolding protein capable of binding numerous partners, including PBPs, FtsA, FtsZ, and GpsB, contributes to the synchronized regulation of septal wall synthesis and Z-ring dynamics [80]. Mutants lacking *ezrA* in *Bacillus subtilis* exhibit phenotypes similar to those deficient in *ponA* (encoding PBP1), characterized by elongated cells and thinner cell wall [98].

(4) DivIVA

DivIVA is a multifunctional scaffold protein that regulates sporulation and cytokinesis in Gram-positive bacteria. Its N-terminal region shares high sequence similarity with GpsB, suggesting a conserved structural and functional relationship between the two proteins [81]. In *Bacillus subtilis* and *Listeria monocytogenes*, DivIVA modulates peptidoglycan synthesis by interacting with bifunctional PBPs [81,99,100].

In *Streptococcus suis*, phosphorylation of DivIVA influences its interaction with the cell wall hydrolase MltG, although it does not directly alter the enzyme’s catalytic activity [82]. Similarly, in *Streptococcus pneumoniae*, DivIVA associates with the hydrolase PcsB and multiple division proteins (FtsZ, FtsA, ZapA, and FtsK), thereby coupling peptidoglycan remodeling with cytokinetic progression [81,82]. Structural analyses reveal that DivIVA specifically targets inwardly curved membrane regions, forming a dumbbell-shaped tetramer with membrane-binding domains at both termini. This configuration enables associated partners to either promote peptidoglycan hydrolysis or facilitate its synthesis, depending on the cellular context [81].

However, existing studies have largely focused on individual proteins or specific bacterial species, leaving a comparative analysis of the interplay between different regulatory systems underexplored. For instance, whether MreB directly participates in division control, and the distinct functional roles of DivIVA and GpsB beyond their apparent redundancy, remain unclear. Moreover, most conclusions are derived from genetic knockout phenotypes, with limited integration of dynamic assembly processes and mechanical feedback mechanisms. Therefore, cross-species, multiscale investigations are urgently needed to critically validate current models.

### 4.5. Protein–Protein Interaction

Peptidoglycan synthesis depends on the concerted action of numerous structural proteins and enzymes, none of which operate in isolation. Its metabolism is primarily governed by protein–protein interactions that modulate enzymatic activity and complex assembly. By inducing conformational changes in their binding partners, these interactions precisely orchestrate cell wall construction through activation or inhibition of enzymatic functions.

*Escherichia coli* contains three bifunctional PBPs (PBP1A, PBP1B, and PBP1C) [101]. To engage these enzymes and modulate their activity, outer membrane lipoproteins (LpoA and LpoB) traverse pores within the cell wall. PBP1A binds LpoA via its outer membrane docking domain (ODD), whereas PBP1B binds LpoA through its UB2H domain. The resulting dimer induces conformational changes in the peptidoglycan synthases PBP1A and PBP1B, activating their transpeptidase activity, which in turn increases the crosslinking of branched pentapeptides and enhances the mechanical strength of the cell wall [83,84]. Formation of these complexes enhances transpeptidase function, increases peptide cross-linking, and strengthens the sacculus by inducing conformational changes in the synthases [102].

Another membrane component, FtsN, binds to the transmembrane region of PBP1B and cooperates with LpoB to accelerate glycan chain polymerization. During cytokinesis, FtsN directly associates with the PBP1B-LpoB complex, regulating septal wall construction through the Tol-Pal system and ensuring synchronized constriction of the outer membrane [85].

Interestingly, some Gram-negative bacteria, such as *Pseudomonas aeruginosa*, lack LpoB homologs. However, Greene et al. identified LpoP, a functional analog that operates via a comparable regulatory mechanism [103]. Acting as a molecular ruler, LpoP constrains the thickness of the sacculus by interacting with inner membrane PBPs through an outer membrane channel whose efficiency depends on pore diameter. As the wall thickens, compression of this conduit reduces Lpo-mediated activation, providing an intrinsic feedback mechanism that balances peptidoglycan synthesis with cell elongation.

FtsW, a transmembrane Lipid II flippase, recruits PBP3 to the division site through its association with the synthases PBP3 and PBP1B [104]. Another essential division factor, FtsN, interacts with multiple components of the divisome, including FtsA, FtsBLQ, ZapA, PBP3, PBP7, FtsW, PBP1B, and FtsQ [85]. Furthermore, stabilization of the dimeric PBPs complex by FtsN enhances its enzymatic activity, thereby promoting efficient peptidoglycan synthesis during cytokinesis [85].

The inhibitory protein IseA regulates DL-endopeptidases, a family of enzymes essential for peptidoglycan remodeling in *Bacillus subtilis* [105]. Tandukar et al. demonstrated that IseA can block the active site of peptidoglycan DL endopeptidase LytE by mimicking its substrate, thereby preventing its overactivation from causing bacterial lysis [106]. It also regulates peptidoglycan degradation through its expression control and inhibitory activity modulation.

In *Escherichia coli*, the periplasmic protease Prc and its outer membrane adaptor NlpI control the abundance of key DD-endopeptidases (MepS and MepH) as well as the lytic transglycosylase MltD [107]. Furthermore, Park et al. identified the periplasmic factor BipP (formerly YhjJ), which mitigates excessive DD-peptidase activity by binding to NlpI and hindering substrate recognition [108]. This interaction provides a homeostatic mechanism that balances peptidoglycan hydrolysis with cell wall expansion.

The above studies reveal a significant difference between two core regulatory logics in bacterial peptidoglycan metabolism: “spatially constrained” feedback and “protein scaffold-based” coordination. Although both rely on protein–protein interactions to achieve regulation, the former emphasizes the constraint of biochemical reactions by the physical barrier of the envelope, whereas the latter focuses on signal integration through soluble protein networks. This distinction suggests that bacterial cell wall homeostasis is not governed by a single mechanism, but rather by a multi-layered homeostatic regulatory architecture composed of spatial structural constraints and molecular interaction networks. A deeper understanding of these two regulatory paradigms not only helps elucidate the fundamental principles underlying bacterial cell wall growth but also provides distinct intervention points for the design of cell wall-targeting antibiotics, ranging from spatially dependent activation pathways (such as the Lpo-PBP interface) to the disruption of key scaffold protein interaction nodes.

## 5. Regulation of Peptidoglycan Homeostasis

Peptidoglycan (PG) homeostasis relies on the concerted and balanced activities of PG synthases and hydrolases. Disruption of this balance compromises cell wall integrity and cell morphology, necessitating the existence of precise regulatory mechanisms that are conserved across different species. Although recent studies have reported such regulatory strategies in *Bacillus subtilis* and *Escherichia coli*, relevant investigations remain relatively limited.

Sassine et al. reported that *ugtP* mutant cells exhibited elevated levels of cell wall precursors, alterations in peptidoglycan architecture, and enhanced dl-endopeptidase activity [109]. Additional deletion of the dl-endopeptidase lytE, which is essential for cell elongation, resulted in pronounced morphological abnormalities. Notably, the *ugtP lytE* double mutant regained its typical rod shape through reduced expression of the peptidoglycan synthase PBP1. These findings suggest that in the absence of *ugtP*, cells must recalibrate the equilibrium between peptidoglycan synthesis and degradation to preserve proper morphology. In *Escherichia coli*, the coordination of peptidoglycan synthesis and hydrolysis is mediated by the FtsEX complex [110]. This membrane-associated ATPase recruits EnvC, an activator of septal hydrolases, into the periplasm and interacts with cytoplasmic FtsA to facilitate the assembly of septal synthases. Du et al. demonstrated that optimal EnvC function requires the ATPase activity of FtsEX [111]. Alcorlo et al. demonstrated that ATP binding by FtsEX in the cytoplasm drives periplasmic conformational changes in EnvC, which lead to the binding and activation of peptidoglycan amidases such as AmiA and AmiB [112].

Furthermore, FtsN contributes to the regulation of septal peptidoglycan (sPG) turnover. It interacts both with denuded glycan (dnG), intermediates of sPG degradation, and with the synthetic complex FtsWIQLB [113]. The SPOR domain of FtsN binds dnG in a cooperative manner, sequestering FtsWIQLB at these degradation sites and thereby preventing further hydrolysis [114]. Conversely, when localized to the sPG synthesis track, FtsN activates the FtsWIQLB machinery. This dynamic redistribution likely functions as a molecular switch, balancing the partitioning of FtsN between degradative and synthetic pathways and ensuring precise coordination of septal construction and remodeling.

Together, these findings reveal that bacterial cell wall homeostasis relies on a multi-level and multi-node cooperative regulatory network. From feedback regulation at the gene–transcription–translation level (e.g., the regulation of pbp1 expression in the ugtP mutant), to functional coupling at the complex level (e.g., the integration of synthesis and hydrolysis by the FtsEX complex), and further to the dynamic allocation between synthesis and degradation pathways via a molecular switch (e.g., FtsN), each layer operates coordinately across distinct spatiotemporal scales. This regulatory architecture goes beyond the traditional linear view centered on a single pathway, emphasizing that the essence of homeostasis lies in dynamic balance and redundant interactions among multiple mechanisms.

## 6. Applications

The structural integrity of peptidoglycan, a fundamental component of the bacterial cell wall, is crucial for cellular viability. Disruption of its synthesis or architecture results in osmotic lysis and cell death, making it an attractive target for antimicrobial development. Two principal classes of clinically employed antibiotics interfere with cell wall biogenesis [115]. (1) Fosfomycin inhibits the cytoplasmic formation of peptidoglycan precursors by blocking uridine diphosphate-N-acetylglucosamine enolpyruvyl transferase (MurA), thereby preventing the initiation of cell wall synthesis. (2) β-lactam antibiotics, including penicillin, disrupt cross-link formation and compromise structural integrity by inactivating PBPs—the enzymes responsible for the final polymerization and cross-linking steps in peptidoglycan assembly. Furthermore, with respect to the processes of peptidoglycan synthesis and hydrolysis, Zhou et al. reported that benzothiazole arylurea derivatives target autolysin-mediated peptidoglycan hydrolases and exhibit potent anti-staphylococcal activity [116]. Kaus-Drobek et al. identified and characterized a novel M23 peptidase, designated StM23, from *Thermus* strain NCTC10353, which displays antibacterial activity against *Listeria monocytogenes* and other Gram-positive bacteria [117]. Regarding transcriptional regulation, Schmidt et al. found that in *Agrobacterium tumefaciens*, the global transcriptional regulator LsrB directly represses the expression of AmpD, a key enzyme involved in peptidoglycan recycling, thereby modulating the AmpR mediated induction of the β-lactamase AmpC. Deletion of *lsrB* results in extreme sensitivity to ampicillin and penicillin [118]. Meanwhile, antimicrobial strategies targeting two-component systems have been systematically reviewed elsewhere and will not be reiterated here [119].Collectively, these studies offer promising new strategies for the design of novel antibacterial agents.

Understanding the regulatory mechanisms of peptidoglycan synthesis provides opportunities to modify cell wall pathways in industrial microbial strains. This mechanistic insight enables targeted genetic modifications to optimize key physiological traits, such as enhancing metabolite transport and secretion, reducing wall rigidity, bolstering tolerance to fermentation-associated stresses, and ultimately improving overall production efficiency. Wu et al. demonstrated that the transcription factor TcsR7 of the *Lactococcus lactis* F44 two-component system binds to the promoter region of *dltD*, a gene involved in D-alanylation of cell wall teichoic acids [120]. Under acidic conditions, tcsR7 expression is upregulated, and TcsR7 activates *dltD* transcription. This regulation increases the positive charge on the cell surface by inducing the dlt operon, thereby enhancing acid tolerance during acid stress. *Corynebacterium glutamicum*, a soil-dwelling species widely used for amino acid production and classified as Generally Recognized as Safe (GRAS), also benefits from such regulatory insights. Liu et al. identified a Y489C substitution in the bifunctional peptidoglycan transpeptidase/glycosidase PonA, which increased the electrocompetence of strain ATCC 13869 by 19.25-fold [121]. This finding provides a foundation for employing targeted genetic modifications to boost industrial yields of glutamate-producing bacteria.

Beyond their biological significance, the distinctive properties of peptidoglycan synthesis and hydrolysis enzymes have driven major technological innovations with far-reaching applications in medical diagnostics and microbiology. First, the use of specific hydrolases, such as fluorescent D-amino acid probes [122], enables dynamic visualization of cell wall assembly at the single-cell level, providing unprecedented spatiotemporal resolution for elucidating the regulatory mechanisms underlying bacterial growth, division, and population behavior. Second, the integration of these enzymes into biosensing platforms has led to a novel strategy for real-time pathogen detection, allowing rapid diagnosis of infectious diseases through in situ lysis that releases diagnostic molecules such as ATP and DNA [123].

## 7. Limitations and Unresolved Questions

Although a substantial body of evidence has accumulated in recent years regarding bacterial peptidoglycan synthesis, hydrolysis, and multi-level regulation, and the basic framework of the relevant molecular pathways and regulatory modules has been preliminarily established, significant knowledge gaps, literature inconsistencies, and model deficiencies remain in this field from the perspectives of systems biology and evolutionary conservation. These limitations hinder a deeper understanding of the nature of cell wall homeostasis and the development of targeted intervention strategies.

Current research remains largely focused on individual regulatory layers, specific proteins, or single model organisms, and thus lacks a systematic dissection of how multi-layered signals are integrated, how cross-talk occurs, and how feedback loops are established. In particular, the temporal coordination and spatial coupling among transcriptional regulation, two-component systems, small RNAs (sRNAs), and scaffold proteins remain poorly supported by quantitative and dynamic evidence. Most existing models still rely on pathway-independent linear descriptions, which fail to capture genuine physiological features such as cross-regulation, redundant compensation, and condition-dependent switching.

At the level of signal perception, the direct activating signals for two-component system stress pathways remain unidentified [124]. The molecular ligands, structural alterations, or physical cues (e.g., membrane tension, cell wall stiffness, lipid II concentration) recognized by systems including WalKR, VxrAB, and CpxA remain at the stage of indirect inference. Discrepancies in the activation cues reported across different studies persist, thereby constraining precise elucidation of the mechanisms underlying cell wall stress responses [125].

Furthermore, certain functional models of key proteins are accompanied by contradictory conclusions. For instance, the capacity of the MreB protein to form polymers remains undisputed; however, its structure, length, and assembly conditions have long been ambiguous and subject to conflicting reports. Using super resolution imaging, Billaudeau et al. discovered that during active growth of *Bacillus subtilis*, MreB assembles into subdiffraction nanofilaments with an average length of less than 200 nm, rather than the micrometer scale long helical fibers previously widely described [126]. The appearance of long fibers was subsequently attributed to an artifact of protein overexpression. This finding directly refutes models proposing that MreB filament length influences the rate of cell wall synthesis, as well as models suggesting that MreB coordinates distal synthetic machineries along the lateral wall. Similarly, the “switch” model of FtsN in septum remodeling has not been fully reproducible across different species or under varying experimental conditions. Bioinformatics analyses reveal that FtsN is a protein of low conservation, with its N terminal region exhibiting considerable variability among distinct species [127]. This low conservation implies that existing models may be species specific or condition dependent, and their general applicability awaits rigorous validation.

Meanwhile, Gram-positive and Gram-negative bacteria exhibit significant evolutionary divergence in their regulatory strategies, yet comparative studies remain severely lacking. The driving factors underlying the differentiation between the two groups in terms of hydrolase inhibition, synthase activation, and spatial assembly are not well understood. Moreover, regulatory differences among opportunistic pathogens, commensals, and industrial strains have not been systematically compared, making it difficult to establish a universal model across bacterial lineages.

## 8. Conclusions and Outlook

Over the past decade, remarkable progress has been made in elucidating the complexity of the bacterial cell wall. Far from being a static protective barrier, the cell wall with peptidoglycan as its principal component, dynamically determines cell shape, size, and mechanical strength, while also orchestrating the fundamental processes of growth and division. In pathogenic species, the cell wall is essential for viability, virulence, and immune evasion, whereas in industrial microorganisms, its integrity and adaptability critically affect fermentation performance and product yield. This review synthesizes recent advances in the regulatory mechanisms governing peptidoglycan synthesis and degradation. Peptidoglycan, a three-dimensional network macromolecule formed by glycan chains cross-linked via peptide bridges, maintains cell wall integrity and function through a fine-tuned balance between its biosynthesis and hydrolysis, a process essential for cellular homeostasis, expansion, and division. Significant progress has been made in elucidating the assembly, activity regulation, and cooperative functions of peptidoglycan biosynthetic enzymes and autolysins. This highly dynamic process is orchestrated by a complex, multi-layered regulatory hierarchy involving transcription factors, two-component systems, non-coding small RNAs, scaffold proteins, and protein–protein interaction networks. Collectively, these mechanisms establish an integrated regulatory system that coordinates processes from gene expression and protein activity to the final assembly of peptidoglycan.

In summary, as knowledge of the core pathways of peptidoglycan metabolism deepens, future research is shifting its focus toward more refined regulatory mechanisms. This includes systematic identification of genomic binding sites for key regulatory proteins to uncover global transcriptional architectures. Progress in this field will depend on deep interdisciplinary integration, fostering synergistic innovation across microbiology, structural and chemical biology, biophysics, and computational sciences, particularly through AI-assisted molecular dynamics simulations and network modeling. Such collaborations efforts are anticipated not only to yield deeper mechanistic insights but also to inspire the design of novel antimicrobial strategies and promote the engineering of high-performance microbial cell factories.

## Figures and Tables

**Table 2:** Downstream Transcriptional Regulation of Peptidoglycan Homeostasis.

Name	Category	Role	Model Organisms	References
Non-coding small RNAs (sRNAs)				
*glmZ*	Non-coding small RNA	Base-pair with glmS mRNA to activate expression for cell wall precursor supply	*E. coli*	[72]
*dsrA*	Non-coding small RNA	Bind to mreB mRNA’s 5′ UTR to reduce MreB levels and alter morphology	*E. coli*	[73]
*dicF*	Non-coding small RNA	Pair with the ftsZ ribosome-binding site to inhibit translation and Z-ring formation	*E. coli*	[74]
*rli27*	Non-coding small RNA	Stabilize lmo0514 mRNA’s 5′-UTR to enhance synthesis for stress resilience	*L. monocytogenes*	[75,76]
proteins				
MreB	Scaffold protein	bridging linkers MreC/D/RodZ to coordinate peptidoglycan synthesis and sense curvature	*B. subtilis* *E. coli*	[77,78]
FtsZ	Cytoskeletal protein	Polymerize into a Z-ring, bind FtsA and ZipA, and recruit MurG for septal synthesis	*E. coli*	[79]
EzrA GpsB	Scaffold protein	Bind PBP1 and FtsZ to coordinate peptidoglycan assembly and Z-ring dynamics	*B. subtilis*	[80]
DivIVA	Scaffold protein	Bind PBPs, PcsB, and FtsZ to couple peptidoglycan remodeling with cytokinesis	*B. subtilis*, *L. monocytogenes*, *S. pneumoniae*	[81,82]
LpoA	Outer membrane lipoprotein	binds to PBP1A via the ODD domain to enhance transpeptidase activity and cross-linking	*E. coli*, *P. aeruginosa*	[83]
LpoB	Outer membrane lipoprotein	Bind to PBP1B’s UB2H domain to strengthen the sacculus via conformational changes	*E. coli*, *P. aeruginosa*	[84]
FtsN	Outer membrane lipoproteins	Bind to PBP1B transmembrane region and cooperate with LpoB to accelerate glycan polymerization via Tol-Pal	*E. coli*	[85]

## Data Availability

No new data were created or analyzed in this study.
